# Effect of transcutaneous electrical nerve stimulation on postoperative pain and fear in children undergoing abdominal surgery: a randomized controlled trial

**DOI:** 10.1590/1806-9282.20251661

**Published:** 2026-08-10

**Authors:** Hilal Kurt Sezer, Bayram Sonmez Unuvar, Metin Gunduz, Sibel Kucukoglu

**Affiliations:** 1 Nigde Omer Halisdemir University, Derbent Campus, Zubeyde Hanim Faculty of Health Sciences, Department of Nursing – Niğde, Turkey.; 2 KTO Karatay University, Faculty of Health Sciences, Physical Therapy and Rehabilitation – Konya, Turkey.; 3 Selcuk University, Faculty of Medicine, Department of Pediatric Surgery – Konya, Turkey.; 4 Selcuk University, Faculty of Nursing, Department of Nursing – Konya, Turkey.

**Keywords:** Abdomen, Acute, Appendicitis, Pain management, Nursing

## Abstract

**OBJECTIVE:**

Pain management in pediatric patients during the postoperative period is crucial for ensuring success in managing the postoperative process. The aim of this study was to determine the effect of Transcutaneous Electrical Nerve Stimulation in pediatric patients undergoing abdominal surgery.

**METHODS:**

This single-center, single-blind randomized controlled trial included pediatric patients aged 7–12 years undergoing abdominal surgery. Participants were randomly assigned to an experimental or control group using stratified block randomization according to the type of surgery (appendectomy or inguinal surgery). The experimental group received Transcutaneous Electrical Nerve Stimulation for 40 min before postoperative mobilization, whereas the control group received routine postoperative care only. Outcome assessors were blinded to group allocation. Pain intensity and fear levels were assessed using the visual analog scale, the Wong-Baker Faces Pain Rating Scale, and the Children’s Fear Scale at baseline and during postoperative mobilization.

**RESULTS:**

As a result of this study, visual analog scale, Wong-Baker Faces Pain Rating Scale, and Children’s Fear Scale mean scores were found to be similar in the experimental and control groups in baseline measurement (p>0.05). The first and second measures visual analog scale, Wong-Baker Faces Pain Rating Scale, and Children’s Fear Scale mean scores were significantly lower in the experimental group than in the control group (p<0.001).

**CONCLUSION:**

The results of the study showed that Transcutaneous Electrical Nerve Stimulation application was effective on pain and fear before postoperative mobilization in pediatric patients undergoing abdominal surgery. Transcutaneous Electrical Nerve Stimulation, a non-pharmacological method, can be used to manage pain and fear in the postoperative period in pediatric patients undergoing abdominal surgery.

**Clinical Trial Registration Number:**

ClinicalTrials.gov Identifier: NCT 05119621

## INTRODUCTION

Epidemiological studies show that more than half (a significant percentage) of children admitted to hospital (49–64%) receive inadequate pain management^1,2^. Remarkable, about 75% of these individuals report pain levels exceeding the threshold of moderate intensity. Pediatric patients are exposed to acute pain due to postoperative and procedural conditions. Even among adult patients, surgical procedures can induce anxiety; however, the experience can be particularly traumatic for children. Pain management in childhood is recommended by both the World Health Organization and pain-focused communities^2,3^. Enabling early mobilization during the postoperative phase holds paramount significance in averting the stagnation of physical functions, muscle deterioration, circulatory complications, and impaired pulmonary capacities^4^. Furthermore, proficiently managing mobilization counteracts the unfavorable physiological repercussions of surgical stress and expedites the postoperative recuperation process. Uncontrolled pain during the postoperative phase remains a prevalent concern among pediatric patients^5^. When pain is not managed effectively, it delays postoperative mobilization, which in turn impairs tissue perfusion. Transcutaneous Electrical Nerve Stimulation (TENS) is an applying that can help relieve acute pain and, when used in combination with opioids, increase the potency of analgesia in chronic pain scenarios. Some studies have investigated the effect of TENS for postoperative pain control in adult patients. Research also shows that TENS can helps adult and pediatric patients experience less acute and chronic pain^6,7^. In a guideline published in 2016, clinicians are advised to incorporate TENS as a supplementary approach to complement other methods of postoperative pain management^8^. There is limited research on the effects of TENS on pain experienced by pediatric patients. This randomized controlled trial aims to determine the effect of TENS in pediatric patients undergoing abdominal surgery.

### Research hypotheses

H_1_: TENS applied before mobilization in the postoperative period reduces the pain level of the intervention group.

H_2_: TENS applied before mobilization in the postoperative period reduces the fear level of the intervention group.

## METHODS

### Study design

**Figure 1:**
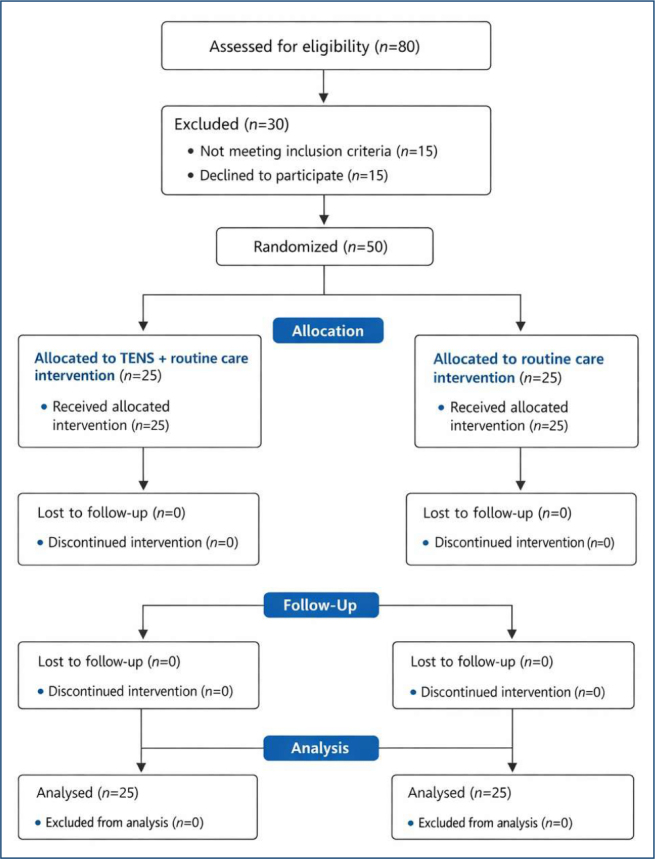
Consolidated Reporting Study Standards flow diagram of participant recruitment, randomization, follow-up, and analysis.

This study is a randomized controlled, single-blind, parallel-group experimental study. The study conducted in the single-center and Consolidated Reporting Study Standards (CONSORT) were followed^9^ (Figure 1).

### Setting and participants

The research was conducted in the pediatric surgery unit of a medical faculty hospital in a provincial center between July 2021–November 2022. A power analysis (3.1 G*Power) was performed to determine the sample size. The results showed that a sample of 40 would be large enough to detect significant differences^10^ (1.1 effect size, 0.05 margin of error, and 0.95 power). The sample consisted of 50 pediatric patients to avoid missing data. The inclusion criteria were (i) being 7–12 years of age, (ii) having undergone abdominal surgery, and (iii) volunteering. The exclusion criteria were (i) receiving analgesic treatment other than the clinic’s routine analgesic administration (opioid or infusion of other painkillers), (ii) having difficulty mobilizing, and (iii) having developed complications during surgery.

### Randomization and blinding

Participants were allocated to the experimental (TENS) or control group using stratified permuted block randomization based on the type of abdominal surgery (appendicitis or inguinal surgery). The randomization sequence was generated by an independent statistician. Allocation concealment was ensured using sequentially numbered, opaque, sealed envelopes prepared according to the randomization table. The envelopes were kept by an independent clinic nurse and opened sequentially by the researcher before the intervention to determine group assignment. Due to the nature of the intervention, participants and the physiotherapist could not be blinded. However, the study was conducted in a single-blind design in which outcome assessors were blinded to group allocation.

### Data collection tools

The data were collected using a personal information form, the Visual Analog Scale (VAS), the Wong-Baker Faces Pain Rating Scale (WB-FACES), and the Children’s Fear Scale (CFS).

Personal Information Form: The Personal Information Form was created by researchers^1,5,6^.

Children’s Fear Scale: The CFS was developed by McMurtry et al. and adapted to Turkish by Gerçeker et al. The instrument assesses pain and anxiety before and after surgery^11,12^.

Visual Analog Scale: VAS is used to turn abstract concepts into numerical values and measure specific dimensions, such as pain. It is a 10- or 100-cm-long horizontal or vertical line (makes no difference) with anchor statements “no pain” at the left-most end and “unbearable pain” at the right-most end. The VAS is an easy-to-use and easy-to-measure scale with no language barrier.

Wong-Baker Faces Pain Rating Scale: The WB-FACES Pain Rating Scale was developed by Wong and Baker in 1981 and revised in 1983. This scale is used to diagnose pain in children aged 3–18 years. Higher scores indicate low pain tolerance^13^.

### Intervention

The research procedure followed a sequential series of steps. First, the researcher provided parents with an explanation of the research’s purpose and the procedure involved. Second, written and verbal consent were obtained from both parents and their respective children. Third, the researcher conducted individual face-to-face interviews with each participant from the experimental group within their rooms. This interview was conducted to complete personal information form prior to mobilization. Fourth, a pretest stage was initiated wherein the parent, researcher, and participant together completed the CFS and WB-FACES. Simultaneously, the participant also completed the VAS as part of the pretest. Fifth, the researcher transitioned to the intervention phase, involving the application of TENS. A specialist in physiotherapy and rehabilitation facilitated this intervention using a TENS device equipped with four electrodes (Compex 3). Placing two electrodes two centimeters to the right of the incision site and the other two centimeters to the left, the specialist configured the following parameters: frequency=100 Hz; pulse width=140 μs; duration=40 min. At the 15th and after the intervention, the researcher administered the CFS, WB-FACES, and VAS as part of the posttest. Following the completion of the posttest, the participant was mobilized immediately. It’s important to note that both the experimental and control groups received the routine postoperative analgesia treatment as part of their care.

Clinic’s Routine Practice: Pediatric patients who undergo abdominal surgery are typically mobilized within 4–6 h after their return to the clinic. Intravenous paracetamol is administered four times within a 24-h period, dosed according to the patient’s weight. The initiation of oral fluid intake commences eight hours after the surgery. Subsequently, if the patient demonstrates tolerance, a gradual transition to light-pureed food is introduced. Once the patient exhibits gas output, the transition to solid food is implemented.

### Ethical considerations

Ethics committee and pharmaceutical and medical devices committee permission were obtained before the study. The study has been registered in the clinical trial database. Permission was obtained from the hospital. Informed consent was obtained from parents and children who agreed to participate in the study.

### Statistical analysis

The primary outcome of the study was postoperative pain intensity measured by the VAS at the first post-test during mobilization. Secondary outcomes included WB-FACES and CFS scores. Data were analyzed using International Business Machines Statistical Package for the Social Sciences Statistics 25. Normality was evaluated with the Shapiro-Wilk test and homogeneity of variance with Levene’s test. Mixed-design ANOVA was used to examine group, time, and group × time interaction effects. Bonferroni correction was applied for multiple comparisons and the significance level was adjusted to p<0.017. Effect sizes were reported as partial eta-squared (η^2^) and interpreted as small (0.01), medium (0.06), or large (0.14).

## RESULTS

It was observed that the children in the TENS and control groups showed a homogeneous distribution (p>0.05; Table 1).

**Table 1:** Sociodemographic characteristics (n=50).

Variable	Category	Experimental (n=25) n (%)/mean±SD	Control (n=25) n (%)/mean±SD	p
Child’s gender	Girl	8 (32)	7 (28)	0.758
	Boy	17 (68)	18 (72)	
Child’s age (year)	Mean±SD	10.24±1.88	9.80±1.76	0.396
Mother’s age (year)	Mean±SD	38.60±4.13	39.12±3.80	0.645
Father’s age (year)	Mean±SD	41.16±4.78	42.40±4.92	0.371
Mother’s education	Primary school	13 (52)	15 (60)	0.202
	High school	9 (36)	10 (40)	
	Bachelor’s or higher	3 (12)	0 (0)	
Father’s education	Primary school	9 (36)	5 (20)	0.182
	High school	12 (48)	20 (80)	
	Bachelor’s or higher	4 (16)	0 (0)	
Family type	Extended	2 (8)	6 (24)	0.123
	Nuclear	23 (92)	19 (76)	
Patient complaints	Yes	21 (84)	19 (76)	0.480
	No	4 (16)	6 (24)	
Type of complaint	Abdominal pain	19 (31)	2 (6)	0.221
	Nausea or vomiting	13 (21)	16 (47)	
	Loss of appetite	12 (19)	9 (26)	
	Distension	18 (29)	7 (21)	
Type of surgery	Appendicitis	20 (80)	20 (80)	0.999
	Inguinal region	5 (20)	5 (20)	
Surgery technique	Open	23 (92)	23 (92)	0.999
	Closed	2 (8)	2 (8)	
Surgery status	Emergency	21 (84)	14 (56)	0.051
	Planned	4 (16)	11 (44)	
Previous surgery history	Yes	9 (36)	9 (36)	0.999
	No	16 (64)	16 (64)	
Previous hospitalization	Yes	9 (36)	10 (40)	0.771
	No	16 (64)	15 (60)	

Values are presented as mean±standard deviation for continuous variables and number (percentage) for categorical variables. Comparisons between groups were performed using Chi-square test for categorical variables and independent samples t-test for continuous variables. No statistically significant differences were observed between the experimental and control groups at baseline (p>0.05). SD: standard deviation.

According to the separate evaluations of the researchers, parents and children, it was determined that the TENS applying reduced the children’s fear level (during and after TENS applying). Based on the researchers’ assessment, a significant time*group interaction effect was observed for CFS scores (F=180.592; p<0.001) and the effect size was determined to be medium. Similar results were obtained in the CFS assessment of parents and children (F=96.000; p<0.001) (F=107.869; p<0.001), (Table 2).

**Table 2:** The distribution of Children’s Fear Scale scores (n=50).

CFS assessment	Time	Experimental (n=25) Mean±SD	Control (n=25) Mean±SD	Between-group (F, p, η^2^)	Group×time (F, p, η^2^)
CFS researcher	Baseline	2.36±0.70^b^	2.44±0.87^b^	F=0.128; p=0.722 η^2^=0.003	F=180.592; p<0.001 η^2^=0.790
	First posttest	0.20±0.41^a^	3.08±1.00^d^	F=174.330; p<0.001 η^2^=0.784	
	Second posttest	0.16±0.37^a^	2.84±0.94^c^	F=178.759; p<0.001 η^2^=0.788	
	Within-group (time)	F=138.363; p<0.001 η^2^=0.855	F=14.607; p<0.001		
			η^2^=0.383		
CFS parents	Baseline	2.92±1.04^c^	2.60±1.04^c^	F=1.185; p=0.282 η^2^=0.024	F=96.000; p<0.001 η^2^=0.667
	First posttest	0.28±0.61^a^	3.00±1.19^c^	F=164.896; p<0.001 η^2^=0.775	
	Second posttest	0.20±0.41^a^	2.84±0.94^c^	F=103.138; p<0.001 η^2^=0.682	
	Within-group (time)	F=110.717; p<0.001 η^2^=0.825	F=2.011; p=0.145 η^2^=0.079		
CFS participants	Baseline	2.40±0.71^b^	2.72±1.02^b^	F=1.659; p=0.204 η^2^=0.033	F=107.869; p<0.001 η^2^=0.692
	First posttest	0.60±0.82^a^	3.40±0.91^d^	F=190.470; p<0.001 η^2^=0.799	
	Second posttest	0.28±0.46^a^	3.24±0.97^d^	F=130.667; p<0.001 η^2^=0.731	
	Within-group (time)	F=109.484; p<0.001 η^2^=0.823	F=11.013; p<0.001 η^2^=0.319		

TS: Test statistics obtained from mixed-model analysis of variance (ANOVA). Between-group comparisons represent differences between the experimental and control groups at each time point. Within-group comparisons (time) indicate changes across measurement times within each group. Group×time represents the interaction effect between group and time. Descriptive statistics are presented as mean ± standard deviation (SD). Different superscript letters (a<b<c<d) within the same row indicate statistically significant differences between time points based on post-hoc comparisons (p<0.05). η^2^: partial eta squared effect size. CFS: Children’s Fear Scale, Baseline: Pre-test, First post-test: 15th minute, Second post-test: 40th minute. CFS: Children’s Fear Scale; SD: standard deviation.

According to the children’s self-measurement, TENS application reduced their pain in the postoperative period (during and after TENS applying). A significant time*group interaction effect was identified for the VAS scores (F=110.723; p<0.001), with the result showing a high effect size. According to the separate evaluations of the researchers, parents and children, it was determined that the TENS applying reduced the children’s WB-FACES scores (during and after TENS applying). Based on the researchers’ assessment, a significant time*group interaction effect was observed for WB-FACES scores (F=122.378; p<0.001), with the effect size categorized as medium (Table 3).

**Table 3:** The Distribution of visual analog scale-Wong-Baker Faces Pain Rating Scale scores (n=50).

Outcome	Time	Experimental (n=25) mean±SD	Control (n=25) mean±SD	Between-group (F, p, η^2^)	Group×time (F, p, η^2^)
WB-FACES (researcher)	Baseline	2.88±0.83^c^	2.16±0.90^b^	F=8.640; p=0.005 η^2^=0.153	F=154.670; p<0.001 η^2^=0.763
	First posttest	0.32±0.63^a^	2.76±0.83^c^	F=140.944; p<0.001 η^2^=0.746	
	Second posttest	0.16±0.37^a^	2.40±0.87^c^	F=137.39; p<0.001 η^2^=0.741	
	Within-group (time)	F=150.287; p<0.001 η^2^=0.865	F=14.234; p<0.001 η^2^=0.377		
WB-FACES (parents)	Baseline	3.68±1.28^c^	2.20±0.76^b^	F=24.593; p<0.001 η^2^=0.339	F=122.378; p<0.001 η^2^=0.718
	First posttest	0.40±0.71^a^	2.44±0.77^b^	F=88.939; p<0.001 η^2^=0.649	
	Second posttest	0.24±0.52^a^	2.36±0.99^b^	F=95.450; p<0.001 η^2^=0.665	
	Within-group (time)	F=134.669; p<0.001 η^2^=0.851	F=0.743; p=0.481 η^2^=0.031		
WB-FACES (participants)	Baseline	2.88±0.93^b^	2.40±0.87^b^	F=3.578; p=0.065 η^2^=0.069	F=66.970; p<0.001 η^2^=0.583
	First posttest	1.08±0.86^a^	2.80±1.08^b^	F=68.097; p<0.001 η^2^=0.587	
	Second posttest	0.56±0.65^a^	2.68±1.11^b^	F=38.723; p<0.001 η^2^=0.447	
	Within-group (time)	F=76.793; p<0.001 η^2^=0.766	F=2.478; p=0.095 η^2^=0.095		
VAS	Baseline	4.68±1.68c	4.12±1.33c	F=1.709; p=0.197 η^2^=0.034	F=110.723; p<0.001 η^2^=0.698
	First posttest	1.72±1.84^a^	4.88±1.48^d^	F=115.075; p<0.001 η^2^=0.706	
	Second posttest	0.72±1.14^a^	4.28±1.21^d^	F=44.819; p<0.001 η^2^=0.483	
	Within-group (time)	F=143.238; p<0.001 η^2^=0.859	F=7.816; p=0.001 η^2^=0.250		

TS: Test statistics obtained from mixed-model analysis of variance (ANOVA). Between-group comparisons represent differences between the experimental and control groups at each time point. Within-group comparisons (time) indicate changes across measurement times within each group. Group×Time represents the interaction effect between group and time. Descriptive statistics are presented as mean±standard deviation (SD). Different superscript letters (a<b<c<d) within the same row indicate statistically significant differences between time points based on post-hoc comparisons (p<0.05). η^2^: partial eta squared effect size. WB-FACES: Wong-Baker Faces Pain Rating Scale. VAS: visual analog scale; SD: standard deviation. Baseline: Pre-test, First post-test: 15th minute, Second post-test: 40th minute.

## DISCUSSION

This randomized controlled trial investigated the effect of pre-mobilization TENS on postoperative pain and fear in children undergoing abdominal surgery. The results demonstrated that children who received TENS experienced significantly lower pain and fear levels during postoperative mobilization compared with those receiving routine care. The large effect sizes observed (e.g., η^2^=0.79 for CFS) suggest that the reduction in fear following TENS application is not only statistically significant but also clinically meaningful. These findings indicate that TENS may be an effective non-pharmacological adjunct for managing postoperative discomfort in pediatric surgical patients.

Postoperative pain in pediatric patients remains an important clinical concern and represents a significant challenge for healthcare professionals, particularly nurses who are responsible for postoperative care and monitoring^14^. Poorly controlled postoperative pain may delay recovery, hinder early mobilization, and increase psychological distress in children, emphasizing the need for effective and safe non-pharmacological pain management approaches in pediatric surgical care.

Previous research has widely documented the effectiveness of TENS in the management of acute and chronic pain, particularly among adult patient populations^6,15^. However, studies examining the role of TENS in pediatric surgical settings remain relatively limited. Earlier investigations have primarily focused on procedural pain or dental interventions in children^16,17,18^. Consequently, evidence regarding the use of TENS for postoperative pain management in pediatric abdominal surgery has remained scarce. In this context, the present study contributes to the existing literature by demonstrating that TENS may reduce both pain and fear associated with postoperative mobilization in pediatric surgical patients.

A recent randomized controlled trial conducted by Li et al. evaluated the effectiveness of transcutaneous electrical acupoint stimulation (TEAS), a technique that combines TENS with acupuncture point stimulation, in children aged 3–15 years undergoing lower extremity orthopedic surgery. The authors reported that TEAS, when applied as part of an enhanced recovery after surgery protocol, significantly reduced postoperative pain levels in pediatric patients^19^. Although TEAS differs slightly from conventional TENS in its application method, both techniques rely on electrical stimulation to modulate pain perception. The findings of the present study are consistent with those reported by Li et al., supporting the potential role of electrical stimulation techniques in pediatric postoperative pain management.

The analgesic effects of TENS are commonly explained by several physiological mechanisms. According to the gate control theory of pain, stimulation of large-diameter afferent nerve fibers can inhibit the transmission of nociceptive signals at the spinal cord level, thereby reducing pain perception^20^. In addition, electrical stimulation may promote the release of endogenous opioids and other neuromodulators that contribute to analgesia^21^. By reducing pain intensity, TENS may also indirectly reduce fear or anxiety associated with postoperative mobilization, particularly in children who may experience heightened distress during the postoperative period.

From a clinical perspective, TENS may represent a practical adjunct to multimodal postoperative pain management strategies in pediatric surgery. The technique is relatively easy to administer, requires minimal training, and involves low equipment costs compared with many pharmacological interventions. Incorporating TENS into postoperative care protocols may therefore help healthcare professionals support early mobilization and improve patient comfort while potentially reducing reliance on pharmacological analgesics.

### Limitations and strengths

This study has several limitations. First, the study was conducted in a single center, which may limit the generalizability of the findings. Second, the study focused only on short-term postoperative outcomes, and no long-term follow-up assessments were performed. One of most significant limitation too of this study is the absence of a sham TENS control group. As TENS produces a distinctive tingling sensation, participants in the experimental group were aware they were receiving an active intervention, and control group participants received no device. This lack of blinding for participants, combined with the lack of a sham device, means that the observed differences in pain and fear scores may be partially attributable to placebo effects. Future studies should incorporate an inert sham TENS device to control for these non-specific effects. Future studies should include multicenter designs with larger sample sizes and incorporate sham-controlled groups to better evaluate the specific effects of TENS. In addition, long-term follow-up studies are needed to determine whether the benefits of TENS extend beyond the immediate postoperative period.

## CONCLUSION

Effective pain management is essential for improving postoperative recovery in pediatric surgical patients. In this randomized controlled trial, TENS applied before mobilization was associated with reduced postoperative pain and fear in children undergoing abdominal surgery. These findings suggest that TENS may be a useful non-pharmacological adjunct for postoperative pain management in pediatric patients. Further randomized controlled studies with larger samples and longer follow-up periods are needed to confirm these findings and better understand the mechanisms underlying these effects.

## Data Availability

The datasets generated and/or analyzed during the current study are available from the corresponding author upon reasonable request.
